# Impact of Sub-Retinal Fluid on the Long-Term Incidence of Macular Atrophy in Neovascular Age-related Macular Degeneration under Treat & Extend Anti-Vascular Endothelial Growth Factor Inhibitors

**DOI:** 10.1038/s41598-020-64901-9

**Published:** 2020-05-15

**Authors:** Jakob Siedlecki, Cheryl Fischer, Benedikt Schworm, Thomas C. Kreutzer, Nikolaus Luft, Karsten U. Kortuem, Ricarda G. Schumann, Armin Wolf, Siegfried G. Priglinger

**Affiliations:** Department of Ophthalmology, Ludwig-Maximilians-University, Munich, Germany

**Keywords:** Biomarkers, Outcomes research

## Abstract

Sub-retinal fluid (SRF) has been discussed as a protective factor against macular atrophy in eyes with neovascular age-related macular degeneration (nAMD).To gauge the impact of SRF on macular atrophy, a database of 310 nAMD eyes was screened for eyes manifesting an SRF-only phenotype under treat & extend anti-VEGF treatment, defined as nAMD expressing CNV exudation beyond the three monthly anti-VEGF loading doses by SRF only without any signs of exudative intra-retinal fluid (IRF) for ≥3 years. Incidence of macular atrophy and treatment responses were evaluated on multimodal imaging, including optical coherence tomography (OCT), blue autofluorescence (BAF) and near-infrared (NIR) confocal scanning laser ophthalmoscopy and fluorescence and indocyanine green angiography (FAG/ICGA). In total, 27 eyes (8.7%) of 26 patients with a mean follow-up of 4.2 ± 0.9 (3–5) years met the inclusion criteria. Mean age was 72 ± 6 (range: 61–86) years. The SRF only phenotype was seen from baseline in 14 eyes (52%), and in 13 eyes (48%) after a mean 1.0 ± 1.3 (1–3) injections. In years 1 to 5, mean 7.5, 5.9, 6.1, 6.1 and 7.0 anti-VEGF injections were given (p = 0.33). Cumulative macular atrophy incidence was 11.5% at year 1, 15.4% throughout years 2 to 4, and 22.4% at year 5. In conclusion, eyes manifesting activity by SRF only in treat & extend anti-VEGF regimen for nAMD seem to exhibit rather low rates of macular atrophy during long-term follow-up. SRF might be an indicator of a more benign form of nAMD.

## Introduction

The introduction of anti-vascular endothelial growth factor (VEGF) therapy in neovascular age-related macular degeneration (nAMD) has improved visual acuity and quality of life for millions of patients worldwide^1^. In the era of anti-VEGF, the long-term maintenance of visual acuity is now challenged less by fibrovascular, and more by atrophic scars^2^.

Incidence and growth of macular atrophy are strongly dependent on CNV activity and resulting anti-VEGF therapy^2^. CNV activity and the need for retreatment are mostly defined by the presence of macular fluid, i.e. intra-retinal fluid (IRF), sub-retinal fluid (SRF), and, less prominently, sub-pigment epithelium fluid^3^. While many studies have shown a robust association of IRF with worsening visual acuity and increasing rates of macular atrophy^4–6^, subretinal fluid presence has paradoxically been shown to correlate with better visual acuity as compared to a completely dry macula, especially if located sub-foveally^7,8^.

The reasons for the documented beneficial effects of sub-retinal fluid on visual acuity are largely unclear. The most common hypothesis concludes that SRF presence reduces the risk of vision-threatening macular atrophy^9^. Therefore, new modified treat & extend regimen tolerating SRF are currently being investigated^10^. However, validating data on the influence of SRF on macular atrophy are lacking - as are reports on the long-term influence on SRF on macular morphology and visual acuity^9^.

After the three anti-VEGF loading doses, a significant proportion of eyes (approximately 11%) exhibit a specific phenotype manifesting CNV activity by SRF only^11^. These eyes represent a unique opportunity to study the effects of SRF on macular atrophy and visual outcomes without the confounding effects of IRF. The aim of this study therefore was to investigate the long-term incidence of macular atrophy and clinical outcomes in eyes presenting with a foveal SRF-only phenotype of nAMD in routine clinical care.

## Methods

### Participants

For this retrospective cohort study, all patients treated with treat & extend anti-VEGF therapy for neovascular AMD at the Ludwig Maximilians-University Munich, Germany between January 2016 and January 2019, were screened for eyes showing recurrent sub-foveal SRF on spectral-domain optical coherence tomography (SD-OCT) during treat & extend therapy. Inclusion criteria for the study were: (I) Absence of intra-retinal fluid (IRF) directly from baseline or after ≤3 loading doses; (II) Fluctuating sub-foveal fluid responsive to anti-VEGF for a duration of ≥ 3 years without significant IRF; (III) Absence of confounding comorbidities (diabetic retinopathy, hereditary retinal disease, diseases of the vitreoretinal interface, status after vitrectomy, optic media opacification impeding sufficient image quality). Institutional review board approval was obtained for this retrospective chart review, and the study adhered to the tenets of the Declaration of Helsinki. All patients provided written informed consent.

Epidemiological data was obtained from each patient, including age, gender, previous ocular comorbidities and procedures, date of first diagnosis of nAMD and anti-VEGF injection, number of anti-VEGF injections, and objective refraction-based early treatment of diabetic retinopathy study (ETDRS) visual acuity at baseline, and throughout years 1 to 5.

### Multimodal imaging

Multimodal imaging was performed as needed at each visit after pupil dilation with topical tropicamide 1% and phenylephrine 2.5%. It included spectral domain optical coherence tomography (SD-OCT) and near-infrared (NIR)/blue autofluorescence (BAF) confocal laser scanning ophthalmoscopy (CSLO) at each visit, and fluorescein (FAG) and/or indocyanine green (ICG) angiography at baseline (all on Spectralis HRA + OCT, Heidelberg Engineering, Heidelberg, Germany).

### Detection of macular atrophy

The presence of macular atrophy was evaluated using multimodal imaging by two experienced readers (JS, CF). In the case of discordance, a third reader was involved to finalize the decision (SP). Screening was performed using BAF and NIR CSLO. In accordance with Lois *et al*.^12^, GA was defined as a reduced signal in both blue autofluorescence (BAF) and near-infrared reflectance (NIR) of >0.05 mm. Due to the confounding effects of hemorrhage, exudate, and blockage of the AF/NIR signal due to the CNV, SD-OCT was used to aid diagnosis in cases of doubt. Adhering to the Consensus Definition for Atrophy Associated with Age-Related Macular Degeneration on OCT and the current literature, atrophy was diagnosed in the presence of choroidal hypertransmission, attenuation of the RPE band or outer retinal atrophy and/or collapse^13–15^.

### Anti-VEGF treatment

Treatment was performed using a treat & extend regimen as described previously^3^. In short, each newly diagnosed CNV lesion was treated with three monthly anti-VEGF loading doses, after which the interval was extended or shortened by two weeks based on the absence of presence of new CNV activity. CNV activity was defined on OCT as (I) any new macular fluid, including SRF, (II) pigment epithelium detachment (PED) increasing central macular thickness >50 µm, or (III) new or increasing macular hemorrhage. On FAG/ICGA, CNV activity was defined as exudation increasing with time. Minimum and maximum interval between injections were 4 and 12 weeks, respectively. In the case of three intervals of 12 weeks without signs of CNV reactivation, treatment was stopped. In the case of CNV reactivation, treatment was again started as in a newly diagnosed lesion. Injected anti-VEGF substances included ranibizumab (Novartis Pharma AG, Basel, Switzerland) and aflibercept (Bayer Healthcare Pharmaceuticals, Berlin, Germany).

### Statistical analysis

All data were gathered and analyzed in Microsoft Excel spreadsheets (Version 16.23 for Mac; Microsoft, Redmond, WA, USA). Statistical analysis was performed in SPSS Statistics 25 (IBM Germany GmbH, Ehningen, Germany). The level to indicate statistical significance was defined as p < 0.05. The Shapiro-Wilk and Kolmogorov-Smirnov tests were employed to test for normal distribution. Statistical analyses of intra-group differences were performed using a dependent two-tailed Student t-test, Mann-Whitney U-test, Chi-Square test, and Fisher’s exact test. A repeated measures ANOVA test was used to compensate for multiple testing, if applicable. Cumulative incidence of macular atrophy was calculated using the Kaplan-Meier method. Pearson’s correlation coefficient was used to test associations of dependent and independent variables. Relevant variables were then included in multiple linear regression analysis. Graphs were plotted in Microsoft Excel showing the standard deviation, if not stated otherwise.

## Results

### Baseline demographics

Out of 310 eyes treated with anti-VEGF inhibitors using a treat & extend regimen between 2014 and 2019, 27 eyes (8.7%) of 26 patients met the inclusion criteria and were included in the analysis. Detailed baseline parameters of the study cohort can be found in Table 1. In brief, mean age was 72.0 ± 6.4 years with a female to male ratio of 18 / 8 (69.2 / 30.8%). Mean total follow-up was 4.2 ± 0.9 (3.0–5.0) years. All eyes (100%) had a minimum follow-up of 3 years, while 59.3% and 48.1% of eyes had a follow-up of 4 and 5 years, respectively.

**Table 1:** Baseline demographic and anti-VEGF treatment characteristics.

No. of eyes (n)	27
No. of patients (n)	26
Gender (m/f)	8/18
Mean age (years)	72.0 ± 6.4 (range: 61–86)
Mean follow-up (y)	4.2 ± 0.9 (3.00–5.00)
**Individual follow-up (n)**	
Year 1	27 (100 %)
Year 2	27 (100 %)
Year 3	27 (100 %)
Year 4	16 (59.3 %)
Year 5	13 (48.2 %)
**CNV (n)**	
Type 1	12 (44.4 %)
Type 2	15 (55.6 %)
SRF only at baseline (n)	14 (51.9 %)
Mean injections until SRF only conversion (n)	1.0 ± 1.3
**Drusen (n)**	
Classic	26 (96.2 %)
SDD	8 (29.6 %)
Sub-retinal hyperreflective material	10 (37.0 %)
**Yearly anti-VEGF injections**	
Year 1	7.5 ± 2.6
Year 2	5.9 ± 3.6
Year 3	6.1 ± 3.3
Year 4	6.1 ± 3.2
Year 5	7.0 ± 2.7
**Sub-retinal fluid presence (n)**	
Baseline	23 (85.2 %)
Year 1	19 (70.3 %)
Year 2	15 (55.5 %)
Year 3	15 (55.5 %)
Year 4	10 (62.5 %)
Year 5	10 (76.9 %)
**Sub-retinal fluid thickness (µm)**	
Baseline	106.3 ± 87.7 (18–297)
Year 1	75.6 ± 71.8 (5–258)
Year 2	60.5 ± 34.6 (22–129)
Year 3	62.1 ± 40.8 (27–183)
Year 4	78.9 ± 44.0 (22–141)
Year 5	50.6 ± 30.6 (15–110)
**Mean BCVA (ETDRS letters)**	
Baseline	64.1 ± 14.0 (40–86)
Year 1	72.3 ± 14.1 (42–86)
Year 2	72.7 ± 13.2 (38–85)
Year 3	72.4 ± 13.8 (40–85)
Year 4	72.7 ± 7.5 (61–83)
Year 5	72.2 ± 9.6 (53–83)
**Atrophy at baseline**	
Yes	1 (3.7 %)
No	26 (96.3 %)

### Therapy adherence

Because of differences in treatment time and follow-up, we performed subgroup analysis of visual acuity to assess for a possible selection bias. Visual acuity in the 11 eyes dropping out early at year 3 (36.1 ± 19.0 letters) vs. the 13 eyes completing year 5 (42.2 ± 9.6 letters) was not statistically different (p = 0.41), making selection bias unlikely.

### Macular and neovascular lesion characteristics

At baseline, 26 eyes (96.2%) showed classic drusen, while subretinal drusenoid deposits (SDD) were present in 8 (29.6) %. Consequently, one eye (3.7%) had SDD only. Twelve eyes (44.4%) had a type 1, 15 eyes (55.6%) a type 2 CNV. No cases of aneurysmal type 1 CNV/polypoidal choroidal vasculopathy or retinal angiomatous proliferation were observed. Of the 12 eyes with a type 1 CNV, 11 (91.6%) had an SRF only phenotype at baseline. Of the 15 eyes with a type 2 CNV, only three (20%) showed the phenotype at baseline due to IRF in 12 eyes (80%). IRF resolution and the resulting conversion into the sub-retinal fluid phenotype was seen after a mean 1.0 ± 1.3 (1–3) injections. Conversely, 79% of eyes with SRF only at baseline had a type 1 CNV. Sub-retinal hyperreflective material (SHRM) was seen in 10 eyes (37.0%).

### Intravitreal anti-VEGF therapy

**Figure 1:**
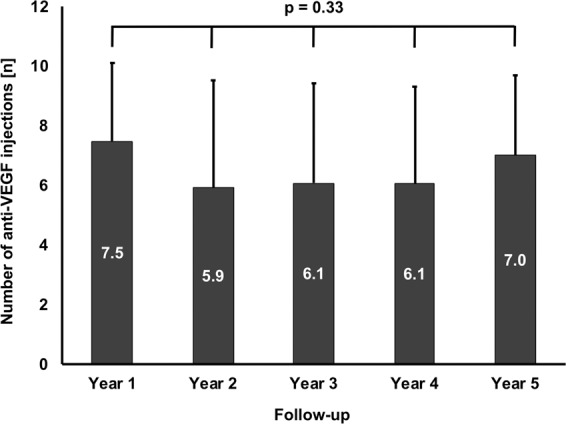
Anti-VEGF treatment during follow-up. Over the span of five years, the mean intravitreal injection frequency remained constant (p = 0.33), starting with 7.5 ± 2.6 injections in year 1, and continuing with 5.9 ± 3.6 in year 2, 6.1 ± 3.3 in year 3, 6.1 ± 3.2 in year 4 and 7.0 ± 2.7 in year 5. Ranibizumab represented 61.0%, aflibercept 39.0% of injections. At least one switch was performed in all patients. (Mean values; error bars: standard deviation).

During the complete follow-up, 715 anti-VEGF injections were given. Of these, 436 (61.0%) were ranibizumab, and 279 (39.0%) were aflibercept. In all patients, at least one switch was performed. Over the span of five years, the mean intravitreal injection frequency remained constant (Fig. 1; p = 0.33), starting with 7.5 ± 2.6 injections in year 1, and continuing with 5.9 ± 3.6 in year 2, 6.1 ± 3.3 in year 3, 6.1 ± 3.2 in year 4 and 7.0 ± 2.7 in year 5.

### Subretinal fluid characteristics

**Figure 2:**
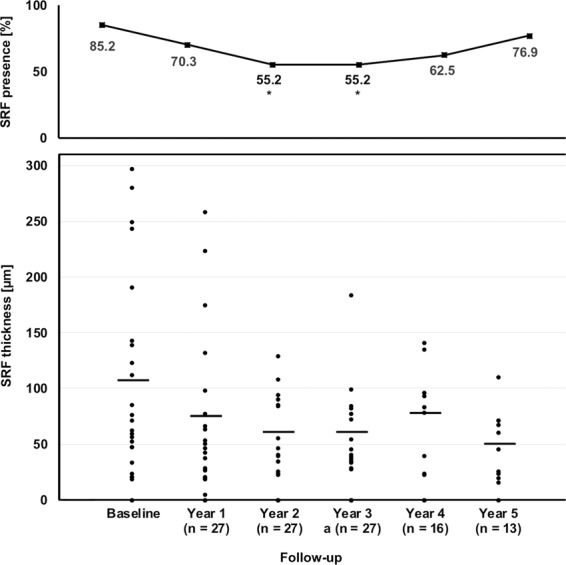
Sub-retinal fluid characteristics during follow-up. Sub-retinal fluid was present in 85.2% of eyes at baseline and 70.3% at year one. There was a significant decrease of SRF presence to 55.5% of eyes at years two and three (p = 0.035), after which it slightly increased again to 62.5% at year four and 76.9% at year five. Maximum sub-retinal fluid thickness showed no significant changes during follow-up with 106.3 ± 87.7 µm at baseline, 75.6 ± 71.8 µm at year 1, 60.5 ± 34.6 µm at year two, 62.1 ± 40.8 µm at year three, 78.9 ± 44.0 µm at year four and 50.6 ± 30.6 µm at year five (p = 0.088).

Sub-retinal fluid was present in 85.2% of eyes at baseline and 70.3% at year one (Fig. 2). There was a significant decrease of SRF presence to 55.5% of eyes at years two and three (p = 0.035), after which it slightly increased again to 62.5% at year four and 76.9% at year five. The average maximum sub-retinal fluid thickness showed no significant changes during follow-up with 106.3 ± 87.7 µm at baseline, 75.6 ± 71.8 µm at year 1, 60.5 ± 34.6 µm at year two, 62.1 ± 40.8 µm at year three, 78.9 ± 44.0 µm at year four and 50.6 ± 30.6 µm at year five (p = 0.088).

### Macular atrophy incidence and prevalence

**Figure 3:**
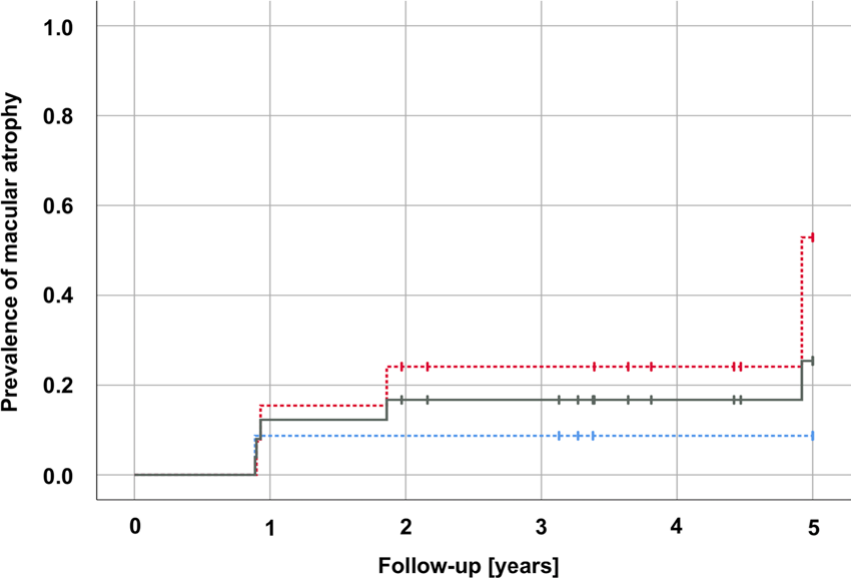
Incidence of macular atrophy during the course of anti-VEGF treatment. Incidence of new macular atrophy was 11.5% at year one, 15.4% throughout years 2–4, and 22.4% at year 5 (grey line). Until year 4, a similar atrophy incidence could be detected in eyes with type 1 and 2 CNV at baseline. At year five, eyes with a type 2 CNV at baseline showed a tendency towards more atrophy than type 1 lesions (p = 0.17).

One eye presented with macular atrophy at baseline (3.7%). Incidence of new macular atrophy in the remaining 26 eyes during follow-up was 11.5% at year one, 15.4% throughout years 2–4, and 22.4% at year 5 (Fig. 3). Until year 4, a similar atrophy incidence could be detected in eyes with type 1 and 2 CNV at baseline. At year five, eyes with a type 2 CNV at baseline showed a tendency towards more atrophy than type 1 lesions (p = 0.17).

No geographic atrophy, i.e. atrophy beyond the CNV borders was seen at baseline or end of follow-up.

### Visual acuity

**Figure 4:**
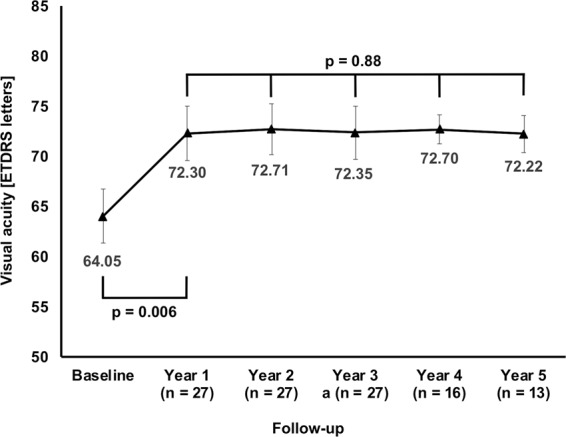
Mean visual acuity during follow up. Starting at 64.1 ± 14.0 ETDRS letters at baseline, visual acuity improved to 72.3 ± 14.1 (42–86) letters at year 1, resulting in a mean visual gain of +8.3 ± 11.9 (−21–30) letters (p = 0.006). Throughout the years 2–5, mean visual acuity remained stable (p = 0.99) and was 72.2 ± 9.6 (23–53) ETDRS letters at end of follow-up. There was no association between the mean SRF thickness during the course of years 1–5 and the change in visual acuity from baseline to last follow-up (r = 0.30; p = 0.13). (Mean values; error bars: standard error of the mean).

Mean visual acuity was 64.1 ± 14.0 (range: 40–86) ETDRS letters at baseline and improved to 72.3 ± 14.1 (42–86) letters at year 1, resulting in a mean visual gain of +8.3 ± 11.9 (−21–30) letters (Fig. 4; p = 0.006). Throughout the years 2–5, mean visual acuity remained stable (p = 0.99) and was 72.2 ± 9.6 (23–53) ETDRS letters at end of follow-up. There was no association between the mean SRF thickness during the course of years 1–5 and the change in visual acuity from baseline to last follow-up (r = 0.30; p = 0.13).

### Adverse events

No serious adverse events (no endophthalmitis, no retinal detachment, and no macular hemorrhage involving the fovea and requiring pneumatic displacement) were observed during the study period.

## Discussion

Along with retinal fibrosis, retinal atrophy is the most frequent cause of severe vision loss secondary to age-related macular degeneration^16,17^. Recent findings suggest that the pathogeneses of atrophy secondary to CNV in neovascular AMD and geographic atrophy as end-stage of dry AMD differ considerably^13,18^. Therefore, the term of ‘macular atrophy’ describing atrophy developing under anti-VEGF therapy for nAMD has been recently suggested to facilitate the distinction against “dry” geographic atrophy^13^. In the era of anti-VEGF, successful fluid management has the greatest impact on long-term macular atrophy and visual acuity, aside from non-modifiable risk factors like age and genetics^9^.

Activity of a CNV and the resulting need of retreatment are nowadays most prominently determined by the presence of any macular fluid on OCT^3,19^. IRF has been found to correlate strongly with incidence of macular atrophy^4,6,9,20^. In contrast, the comparison of age-related macular degeneration treatment trials (CATT) have surprisingly found that eyes with SRF reproducibly showed better visual acuity than their completely dry counterparts^21^. Furthermore, smaller retrospective studies found persisting SRF to be compatible with good long-term visual outcomes, possibly owing to protective effects against macular atrophy^8^. Therefore, new treatment approaches are beginning to tolerate SRF in treat & extend or pro re nata-regimen, while validating long-term data on the impact of SRF on macular anatomy are still lacking.

In this context, our study provides the to our knowledge first data on the anatomic and functional outcomes in eyes manifesting a “sub-retinal fluid only” phenotype of neovascular AMD, defined as neovascular lesions showing activity with recurrent SRF responsive to anti-VEGF only, with a complete absence of IRF after three anti-VEGF loading doses. In our study, only 22.4% of eyes had developed new macular atrophy by 5 years, which can be interpreted as surprisingly low. In the CATT trial, new macular atrophy was seen in 38% of eyes after five years using color fundus photography and fluorescence angiography^22^. In studies using OCT and BAF CSLO, incidence rates are usually even higher, for example 42% to 61% at year 2 in studies by Mantel I *et al*.^23^ and Schütze C *et al*.^24^, 54% at year 4 in a recent study by Li *et al*.^25^, or 54.2% at year 5 in a treat & extend setting essentially identical to our study (Berg *et al*.)^26^.

Interestingly, all cases of atrophy in our study had a close topographic relationship with the CNV lesion. These findings correlate well with recent studies showing that eyes treated with anti-VEGF therapy for neovascular AMD are most likely to develop atrophy in areas topographically corresponding to the CNV lesion, and unlikely to develop de-novo geographic atrophy beyond the CNV area^13,14,18^. Therefore, a strong relationship between CNV toxicity, conferred by direct infiltration of the retina by fibrovascular tissue and exudation, can be assumed – which, conversely, stresses the potential importance of foveal SRF, if proven protective.

In this context, the presence of SRF represents the most probable explanation for the low incidence of macular atrophy of 22.4% at year five in our study. On one hand, SRF might be a protective factor by itself, with popular hypotheses including that SRF might act as a buffer against the direct toxic effects of the CNV or the diseased RPE, or contain certain neuroprotective factors promoting RPE and outer retinal survival^9^. On the other hand, SRF might be a biomarker for a more benign disease associated with type 1 CNV acting as a trophic support to the outer retina, especially in the absence of IRF or dynamic, aggressive pigment epithelium detachments (PED)^27–29^. Recently, Christenbury *et al*. found that macular atrophy progresses significantly slower in areas corresponding with pigment epithelium detachments containing neovascular type 1 CNV flow on OCT-A^30^. Accordingly, the presence of SRF only might be a good indicator of successful CNV management, as CNV infiltration of the retina and IRF are prevented by the proactive treat & extend regimen, thus forcing type 2 lesions back below under the RPE, where such type 1 CNVs can persist in a quiescent, non-infiltrative, trophic state below the RPE. Recently, Nakano *et al*.^31^ found that type 1 CNV show significantly lower vessel junction densities than type 2 CNV on OCT-A, suggesting type 1 CNV to represent more mature vessels. Due to the associated pericyte coverage, mature type 1 CNV are less prone to degradation by VEGF inhibition^32^. In this context, it might be beneficial to adjust anti-VEGF dosing to prevent CNV type 1 into type 2 conversion, while the persistence of supportive type 1 CNV and their biomarker SRF might be tolerable. This hypothesis is reinforced by the fact that there was no influence of baseline CNV type on the long-term incidence of atrophy in our study.

In a clinical context, the tolerance of stable SRF offers an opportunity to partially alleviate the present anti-VEGF treatment burden for patients and health-care professionals. Recently, the FLUID study investigating a modified treat & extend regimen tolerating SRF found a non-inferior gain in ETDRS letters at year 2 with significantly less (15.8 vs. 17) anti-VEGF injections as compared to a strict protocol drying all types of fluid^10^. In contrast to the FLUID study, our protocol did not tolerate, but eliminated sub-retinal fluid. Nevertheless, our present retrospective study provides low rates of long-term atrophy with very good visual gains (8.3 letters on average at year 1) maintained over 5 years, which is comparable to superior to other long-term studies using treat & extend regimen, e.g. Andrean *et al*.^33^ reporting a gain of 9.7 letters after 50 injections, or Berg *et al*.^26^ reporting a gain of 3.4 letters at year 5. In this respect, SRF might be interpreted more as a biomarker of efficient disease management preventing IRF and bleeding, and less as a by itself protective factor. On the contrary, the sole appreciation of visual acuity might represent an only partial view on visual function; several studies have recently shown that sub-retinal fluid significantly reduces retinal sensitivity, which might impair visual performance especially under low-luminance^34,35^.

Several limitations to our study can be found. In addition to its retrospective nature, our study has a limited sample size, which is also affected by dropout during follow-up. Moreover, we lack a control group of eyes showing activity by both SRF and IRF to undermine our hypothesis and to show that these eyes with SRF and IRF, treated by the same standards, show more atrophy. Further studies, including randomized clinical trials, are warranted.

In conclusion, eyes manifesting neovascular activity by sub-retinal fluid only in a treat & extend anti-VEGF regimen for neovascular AMD seem to exhibit rather low rates of long-term atrophy and stable visual outcomes even during long-term follow-up. Even if limited by a small sample size, these data support further research into the tolerability of subretinal fluid in anti-VEGF therapy. Further prospective studies with in-depth analyses of the connection between sub-retinal fluid and macular atrophy are needed for final evidence.
